# A plasma SNORD33 signature predicts platinum benefit in metastatic triple-negative breast cancer patients

**DOI:** 10.1186/s12943-022-01504-0

**Published:** 2022-01-18

**Authors:** Biyun Wang, Yannan Zhao, Yi Li, Yingying Xu, Yun Chen, Qiuyu Jiang, Dingjin Yao, Li Zhang, Xichun Hu, Chaowei Fu, Si Zhang, She Chen

**Affiliations:** 1 Department of Medical Oncology, Fudan University Shanghai Cancer Center, Shanghai Medical College, Fudan University, 270 Dong’an Road, Xuhui District, Shanghai, 200032 China; 2 NHC Key Laboratory of Glycoconjugate Research, Department of Biochemistry and Molecular Biology, School of Basic Medical Sciences, Fudan University, 130 Dong’an Road, Xuhui District, Shanghai, 200032 China; 3 School of Public Health, Key Laboratory of Public Health Safety, NHC Key Laboratory of Health Technology Assessment, Fudan University, Shanghai, China; 4 Department of Gastroenterology and Hepatology, Shanghai Institute of Liver Diseases, Zhongshan Hospital, Fudan University, Shanghai, China

**Keywords:** Breast cancer, Platinum, Prognostic biomarker, SNORD33, MeCP2, Chemoresistance

## Main text

Breast cancer is the most common cancer in women [1]. Triple-negative breast cancer (TNBC), defined by the absence of estrogen receptor (ER), progesterone receptor (PR) and ErbB2 receptor (HER2), accounts for 10–20% of all invasive breast cancers [2]. TNBC has limited treatment options, is prone to recurrence and metastasis, and has a poor prognosis [3]. Platinum has been proved efficacious and is widely used in the neoadjuvant and metastatic treatment of TNBC [4]. However, many patients are refractory to platinum-based regimens and quickly relapse. Here, we aimed to identify a reliable, robust and convenient liquid biopsy approach for rapid prediction of platinum response in TNBC patients.

**Fig. 1:**
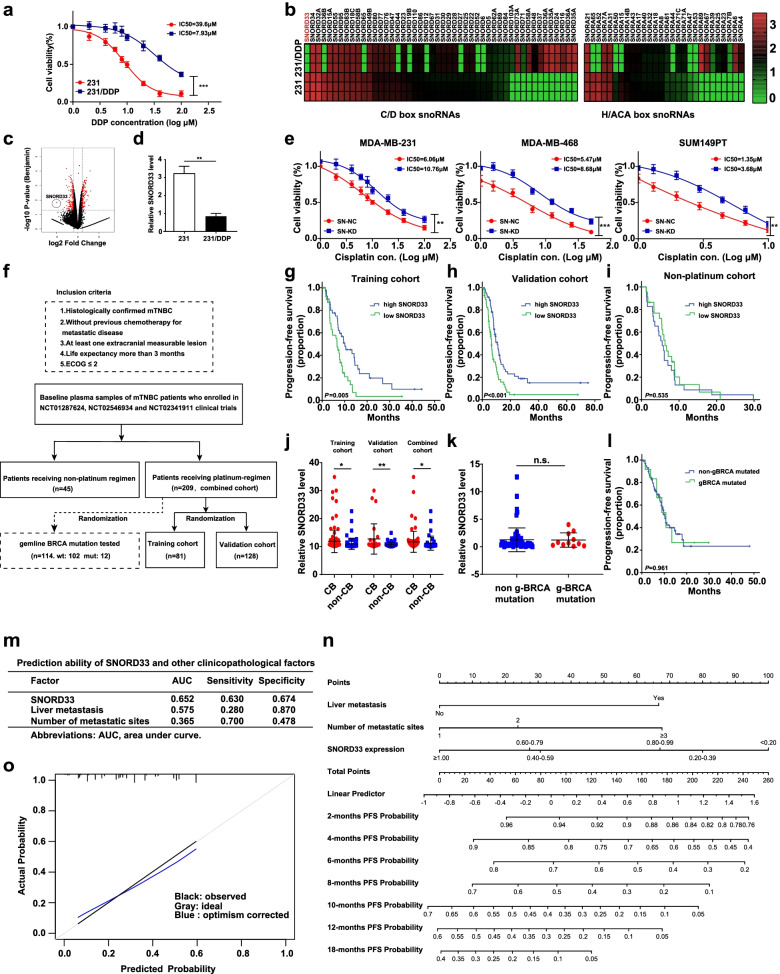
Reduced SNORD33 level predicts cisplatin (DDP) resistance in mTNBC. **a** 231/DDP cells are resistant to cisplatin. 231 and 231/DDP cells were incubated with indicated concentration of DDP for 48 h. CCK8 assays were performed to detect cell viability. *n* = 3; *** represents *P* < 0.001; two-tailed t-test. **b** Heat map analysis displays the differential expression of snoRNAs including SNORD33 in 231 and 231/DDP cells. **c** Volcano map analysis shows that SNORD33 expression is most significantly reduced in 231/DDP cells. **d** SNORD33 is down-regulated in 231/DDP cells. SNORD33 levels in normal 231 cells and cisplatin-resistant 231/DDP cells were assessed by qRT-PCR and normalized against U6. *n* = 3; ** represents *P* < 0.01; two-tailed t test. **e** SNORD33 knockdown increases cell viability of cisplatin treated TNBC cells. SNORD33 was knocked down in MDA-MB-231, MDA-MB-468 and SUM149PT cells. Cells were then treated with cisplatin at indicated concentrations for 48 h and cell viability was determined by CCK8 assay. *n* = 3; ** represents *P* < 0.01, *** represents *P* < 0.001; two-tailed t test. **f** Patients selection and study design: plasma from patients (*n* = 209) who received platinum-based regimens in NCT01287624, NCT02341911 and NCT02546934 trials were included in platinum-regimen cohort and those (*n* = 45) who received non-platinum regimen were included in non-platinum-regimen cohort. Peripheral blood mononuclear cells from patients (*n* = 114) in platinum-regimen cohort were randomly tested for gBRCA mutation. **g**, **h** Kaplan–Meier survival curves for median progression-free survival (PFS) in mTNBC patients receiving first-line platinum-based regimen based on the expression of SNORD33. Training cohort (*n* = 81), *P* = 0.005 (**g**); validation cohort (*n* = 128), *P* < 0.001 (**h**). Cut-off threshold was median value in these cohort; log-rank test. **i** Kaplan-Meier survival curves for median PFS in mTNBC patients received first-line non-platinum-based regimen. Cut-off threshold was median value in this cohort, *P* = 0.053; log-rank test. **j** Plasma SNORD33 level was significantly higher in patients reaching clinical benefit (CB, CR + PR + SD>6 months). Training cohort (11.88 versus 10.98, *P* = 0.012), validation group (11.72 versus 11.23, *P* = 0.006), combined cohorts (11.88 versus 10.98, *P* = 0.038); two-tailed t test. **k**, **l** BRCA mutation neither correlates with plasma SNORD33 level (1.27 versus 1.22, *P* = 0.949) (**k**), nor relates to PFS (**l**) in patients with platinum-based chemotherapy. Kaplan-Meier survival curves for median PFS in mTNBC patients received first-line non-platinum-based regimen based on gBRCA mutation (**l**). Cut-off threshold was median value in this cohort (*n* = 114). *P* = 0.961; log-rank test. **m** The prognostic accuracy for platinum response by the SNORD33 signature, liver metastasis and number of metastatic sites. **n** Prognostic nomogram to assign the probability of PFS for TNBC patients after first-line platinum treatment initiation. The probability of PFS at 2, 4, 6, 10, 12 and 18 months can be obtained as function of total points calculated as the sum points of each specific variable. Points are assigned for each risk factor by drawing a line upward from the corresponding values to the ‘point’ line. The total sum of points for three risk factors is plotted on the ‘total points’ line. A vertical line is drawn for reading the corresponding predictions of 2, 4, 6, 10, 12 and 18 months PFS probability. **o** A calibration curve of the nomogram. The calibration of the prediction model was performed by a visual calibration plot comparing the predicted and actual probability of PFS. The 1000 bootstrap resamples for internal validation was used to assess the predictive accuracies of nomogram

First, we generated a cisplatin (DDP)-resistant TNBC cell line, MDA-MB-231/DDP, which is 5-fold more resistant to cisplatin than its parental line MDA-MB-231 (estimated IC50 values: 39.6 μmol/L versus 7.93 μmol/L) (Fig. 1a). A total of 277 RNA were identified to be aberrantly expressed in 231/DDP cells using RNA-seq analyses (Fig. 1c and S1a). Interestingly, small nucleolar RNAs (snoRNAs) showed the highest variation rate (12.3%), compared with other groups, such as mRNA, lncRNAs and miRNAs (Fig. S1a, right panel). snoRNAs are non-coding RNA (ncRNA) molecules of ~ 60–300 nucleotides in length and can be detected in plasma or serum [5]. The amount of snoRNAs in blood indicates different disease status and associates with clinicopathological features and prognosis [6–9]. Compared with miRNA, ctDNA and exosome, snoRNAs are more stable, technically easy to enrich and detect, therefore, more suitable to serve as prognostic biomarkers [7, 10–13]. We then validated the expression of snoRNAs using qRT-PCR. Among all changed snoRNAs, SNORD33 showed the largest fold (32.2) of change (Fig. 1c), and with relative high abundance (Fig. 1b-d). The antisense oligonucleotide (ASO2) successfully silenced SNORD33 expression in MDA-MB-231, MDA-MB-468 and SUM149PT cells (Fig. S1b). SNORD33 knockdown reduced cisplatin-induced cell death, as measured by CCK-8 assay (Fig. 1e) and colony formation assay (Fig. S2a). Furthermore, a significantly decreased apoptotic cells (Fig. S2b), along with significantly increased level of anti-apoptotic proteins (Mcl-1 and Bcl-2) and reduced expression of pro-apoptotic proteins (cleaved caspase-3 and caspase-9), were observed in SNORD33 knockdown cells upon cisplatin treatment (Fig. S2c, d), indicating that reduced SNORD33 promotes cisplatin resistance in TNBC cells.

Considering the potential of SNORD33 as a liquid biopsy biomarker, we investigated the association between the baseline plasma expression of SNORD33 and corresponding prognosis in mTNBC (metastatic triple-negative breast cancer) patients who received first-line platinum-based chemotherapy (Fig. 1f). In the training cohort, 81 patients were divided into high-SNORD33 and low-SNORD33 expression groups according to the median 2-ΔΔCt value of SNORD33 expression in baseline plasma. Baseline characteristics were well-balanced between these two groups (Table S1). As shown in Fig. 1g, low-SNORD33 group had significantly shorter progression-free survival (PFS) than high-SNORD33 group (6.6 versus 10.1 months; *P* = 0.005). Consistently, in both validation cohort of 128 samples (Fig. 1h) and the combined cohort (Fig. S3a), low-SNORD33 group showed significant worse PFS compared with high-SNORD33 group (validation cohort: 6.5 versus 10.1 months; *P* < 0.001; combined cohorts: 6.6 versus 10.1 months; *P* < 0.001). Likewise, low-SNORD33 group showed significantly reduced overall survival (OS) compared with high-SNORD33 group (22.2 versus 40.6 months; *P* = 0.016, Fig. S3b) in the combined cohort. Univariate Cox regression analysis showed that low SNORD33 expression, number of metastatic sites, visceral metastasis and liver metastasis were significantly associated with reduced PFS and OS in the combined cohort (Table S2). In the multivariate analysis, the signature of SNORD33 was an independent predictive factor of the PFS with platinum-based chemotherapy in the combined cohort (Table S2). Plasma SNORD33 was significantly higher in patients reaching clinical benefit (CB) (defined as complete response (CR), partial response (PR) and stable disease (SD)>6 months) compared with those who failed to reach CB in the training cohort (11.88 versus 10.98, *P* = 0.012), validation group (11.72 versus 11.23, *P* = 0.006) and combined cohorts (11.88 versus 10.98, *P* = 0.038) (Fig. 1j). Furthermore, Kaplan-Meier survival curves from patients treated with non-platinum chemotherapy (gemcitabine plus paclitaxel) (Table S3) revealed that variation in baseline plasma SNORD33 profile did not associate with PFS (5.5 versus 6.5 months; *P* = 0.535, Fig. 1i), indicating that the predictive value of SNORD33 expression is platinum-specific. BRCA mutation status, the most studied biomarker for platinum sensitivity in breast cancer, did not correlate with plasma SNORD33 level (Fig. 1k), nor related to PFS in patients with platinum-based chemotherapy (Fig. 1l).

ROC test was used to evaluate the value of SNORD33 in predicting PFS in mTNBC patients treated with platinum-based regimens in the combined cohort. SNORD33 signature showed the strongest predictive value (AUC = 0.652) for PFS, compared with other single clinicopathological risk factors (liver metastasis, AUC = 0.575; number of metastatic sites, AUC = 0.365), suggesting better performance of SNORD33 signature as a surrogate predictor for platinum-based chemotherapy outcome (Fig. 1m). Based on the estimated coefficients in the multivariate Cox regression model included SNORD33 expression, liver metastasis and number of metastatic sites, we then built a prognostic nomogram to assign PFS probability at 2, 4, 6, 10, 12 and 18 months after first-line platinum treatment. In this nomogram, the point system work by ranking the regression coefficients, with SNORD33 expression converted into 100 points, followed by number of metastatic sites and liver metastasis (yes/no). The final PFS probability was calculated by adding up the score of each item using the nomogram depicted in Fig. 1n. The prediction performance of the nomogram was assessed by the discrimination and calibration. The concordance index (C index), estimating the discriminative ability of the nomogram, was 0.664. A calibration curve also showed high consistency between the predicted and observed PFS probability (Fig. 1o). This nomogram can predict PFS possibility upon platinum-based treatment in different months, information that could be factored in during regimen selection.

Furthermore, a decrease of cisplatin-induced cell toxicity was observed in SNORD33 knocked-down lung adenocarcinoma and urinary bladder carcinoma cell lines (Fig. S4). In 50 metastatic NSCLC patients treated with first-line cisplatin/carboplatin-based regimens, low baseline plasma SNORD33 group had significantly shorter PFS (7.7 versus 15.5 months; *P* = 0.023) than high-SNORD33 group (Table S4, Fig. S5a). And plasma SNORD33 was significantly higher in patients reaching CB compared with those who failed to reach CB (1.36 versus 0.34, *P* = 048, Fig. S5b). The signature of SNORD33 was an independent predictive factor of the PFS with first-line cisplatin/carboplatin-based regimens in lung cancer (Table S5). These data suggest SNORD33 might serve as a feasible predicting biomarker for platinum.

**Fig. 2:**
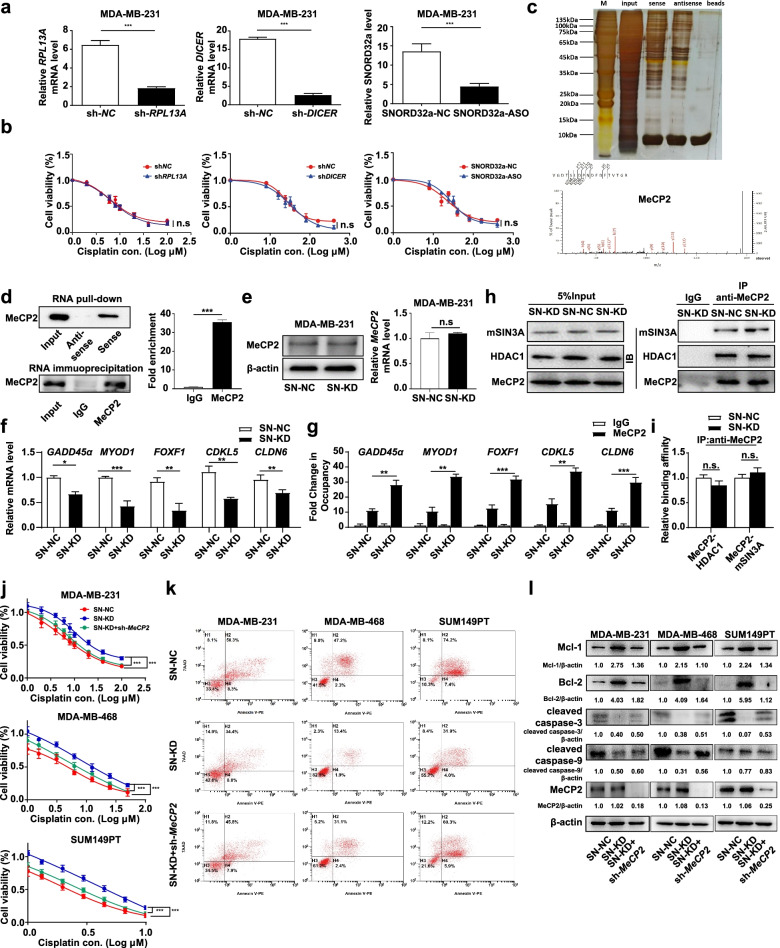
MeCP2 contributes to SNORD33 mediated cisplatin resistance in mTNBC. **a***RPL13A*, *DICER* enzyme, or SNORD32a were knocked down in MDA-MB-231 cells. **b***RPL13A*, *DICER* enzyme, or SNORD32a knockdown do not change the cell viability in response to platinum. These results indicated that platinum resistance arising from loss of SNORD33 may be not caused by alterations of host gene, forming functional miRNA or rRNA modification. **c** The silver-stained bands showed the bands of the sense and antisense strands. The different bands were concentrated in the range of 50–80 kDa and 30–40 kDa. Mass spectrometry show that MeCP2 is a candidate protein for SNORD33 interaction. **d** MeCP2 immunoprecipitates were enriched by SNORD33. RNA pull-down (upper panel) and RIP (RNA immunoprecipitation) assays (lower panel) were performed in MDA-MB-231 cells. Anti-IgG was used as a negative control. *n* = 3; *** represents *P* < 0.001; two-tailed t test. **e** SNORD33 knockdown in MDA-MB-231 cells doesn’t alter *MeCP2* mRNA and protein levels. **f** mRNA levels of MeCP2 target genes are decreased in MDA-MB-231 cells knocking down of SNORD33. The mRNA levels of *GADD45α*, *MYOD1*, *FOXF1*, *CDKL5*, *CLDN6* in MDA-MB-231 cells were determined by qRT-PCR. *n* = 3; * represents *P* < 0.05, ** represents *P* < 0.01, *** represents *P* < 0.001; two-tailed t test. **g** SNORD33 knockdown promotes MeCP2 binding to downstream target gene promoter. SNORD33 was knocked down in MDA-MB-231 cells and chromatin immunoprecipitation (ChIP) assay was performed. qRT-PCR quantification of the immunoprecipitated DNA were measured. Normal rabbit IgG were used as a negative control. Values represented enrichment relative to input DNA. Data are presented as mean ± SD; ** represents *P* < 0.01, *** represents *P*<0.001; one-way ANOVA. **h**, **i** SNORD33 knockdown does not change the binding of co-repressor mSIN3A and HDAC1 to MeCP2. SNORD33 was knocked down in MDA-MB-231 cells and co-immunoprecipitation was performed by MeCP2 antibody. MeCP2, mSIN3A and HDAC1 proteins were detected with western blot (**h**). The binding affinity between MeCP2/mSIN3A and MeCP2/HDAC1 was quantified (**i**). *n* = 3; n.s. represents not significant; two-tailed t test. **j** Down-regulation of MeCP2 rescues SNORD33 knockdown induced cell death. The proliferation of cisplatin treated MDA-MB-231, MDA-MB-468, SUM149PT cells was determined by CCK8 assay. *n* = 3; *** represents *P* < 0.001; two-tailed t test. **k**, **l** Down-regulation of MeCP2 rescues SNORD33 knockdown decreased cell apoptosis (**k**) and induced alteration of apoptotic markers (**l**). Apoptosis of cisplatin treated cells was determined by flow cytometric analysis. Western blot was used to detect the indicated apoptotic markers in cisplatin treated cells. 10 μM cisplatin for MDA-MB-231, 8 μM cisplatin for MDA-MB-468 and 3 μM cisplatin for SUM149PT cells

We next investigated the mechanisms underlying SNORD33 affected platinum sensitivity. *SNORD33* locates in intron 4 of host gene ribosomal protein L13a (*RPL13A*) [14]. It participates in the 2-O′- ribose methylation of 18S rRNA [14]. Does SNORD33 regulate cisplatin resistance by i) its host gene; ii) sliced into functional miRNA; iii) rRNA modification and subsequent protein translation? We knocked down *RPL13A* (host gene), *DICER* enzyme (an endoribonuclease), or *SNORD32a* (homologue to SNORD33 modifying 18S rRNA during cell proliferation) in MDA-MB-231 cells (Fig. 2a), and found that the cell viability in response to platinum was not changed (Fig. 2b). We then employed RNA-pull down assay combined with mass spectrometry to identify SNORD33 binding proteins. Methyl-CpG-binding protein 2 (MeCP2), an important member of the methyl-CpG-binding domain (MBD) family with epigenetic control functions [15], was identified as a potential target to convey SNORD33 drug resistance (Fig. 2c, d and Fig. S6a). The interaction between SNORD33 and MeCP2 was further confirmed by RNA immunoprecipitation (RIP) assays (Fig. 2d). The knockdown of SNORD33 in MDA-MB-231 cells did not alter mRNA and protein levels of MeCP2 (Fig. 2e), but reduced mRNA levels of most MeCP2 target genes, including *GADD45α*, *MYOD1*, and *CLDN6*, which promotes cell apoptosis (Fig. 2f). MeCP2 can repress target-gene expression by binding to CpG-methyl groups on DNA via its MBD domain [16]. In another model, MeCP2, via recruiting mSIN3A and HDAC1, induces histone deacetylation, chromatin compaction, resulting in gene repression [16] (Fig. S6b). We observed that SNORD33 knockdown promoted MeCP2 binding to target gene promoter in MDA-MB-231 cells by chromatin immunoprecipitation (ChIP) assay (Fig. 2g), while the formation of MeCP2/mSIN3A/HDAC1 complex was not affected (Fig. 2h-i). Therefore, SNORD33 may regulate MeCP2 targeted gene expression through disrupting its binding with methylated CpG DNA (Fig. S6b). We then knocked down *MeCP2* in SNORD33-knockdown MDA-MB-231, MDA-MB-468 and SUM149PT cell lines (Fig. S6c). Down-regulation of MeCP2 expression can partially reverse the platinum resistance of TNBC cell lines caused by loss of SNORD33, as measured by CCK-8 assay (Fig. 2j) and colony formation assay (Fig. S7). Consistently, MeCP2 down-regulation attenuated SNORD33-knockdown induced decreased apoptotic cells and pro-apoptotic proteins, and increased anti-apoptotic proteins (Fig. 2k, l). Together, these data imply that MeCP2 contributes to SNORD33 mediated platinum resistance in TNBC cells.

## Conclusions

Together, we identified plasma SNORD33 signature as a predictor for the sensitivity of platinum-based chemotherapy in mTNBC patients. Furthermore, we showed that MeCP2 could convey SNORD33 associated platinum resistance. Our findings may have immediate translational relevance for precision platinum-based chemotherapy.

## Supplementary Information

**Additional file 1.** Methods.

**Additional file 2: Supplementary Figure 1.** Aberrantly expressing RNA in 231/DDP cells. **Supplementary Figure 2.** SNORD33 knockdown increases proliferation and decreases apoptosis of TNBC cells. **Supplementary Figure 3.** Reduced SNORD33 level is correlated with poor prognosis of mTNBC patients who received first-line platinum-based chemotherapy. **Supplementary Figure 4.** Increased cell viability was observed in cisplatin treated cell lines with SNORD33 knockdown. **Supplementary Figure 5.** Reduced SNORD33 level correlates with poor prognosis of non-small cell lung cancer (NSCLC) patients who received first-line platinum-based chemotherapy. **Supplementary Figure 6.** MeCP2 is a candidate protein binding with SNORD33. **Supplementary Figure 7.** Down-regulation of MeCP2 partially rescues SNORD33 knockdown increased cell colony formation. **Supplementary Figure 8.** Down-regulation of MeCP2 rescues SNORD33 knockdown decreased cell apoptosis and induced alteration of apoptotic markers.

**Additional file 3: Supplementary Table 1.** Baseline characteristics in mTNBC patients received first-line platinum-containing regimens. **Supplementary Table 2.** Univariate and multivariate analysis of prognostic factors associated with progression-free survival and overall survival in the combined cohort. **Supplementary Table 3.** Baseline characteristics in mTNBC patients received first-line non-platinum-containing regimens. **Supplementary Table 4.** Baseline characteristics in lung adenocarcinoma patients received first-line platinum-containing regimens. **Supplementary Table 5.** Univariate and multivariate analysis of prognostic factors associated with progression-free survival in lung adenocarcinoma patients.

**Additional file 4.**

## Data Availability

Original RNA sequencing and mass spectrometry data is available from the corresponding author upon reasonable request.

## Abbreviations

ASO: Antisense oligonucleotides
AUC: Area under the curve
Bcl-2: B-cell lymphoma-2
CI: Confidence interval
CLDN6: Claudin 6
ER: Estrogen receptor
GADD45α: Growth arrest and DNA damage inducible alpha
HER2: ErbB2 receptor
HR: Hazard ratio
MBD: Methyl-CpG-binding domain
Mcl-l: Myeloid cell leukemia-1
MeCP2: Methyl-CpG-binding protein 2
mTNBC: Metastatic triple negative breast cancer
MYOD1: Myogenic differentiation 1
ncRNA: Non-coding RNA
OS: Overall survival
PFS: Progress-free survival
PR: Progesterone receptor
RIP: RNA immunoprecipitation
ROC: Receiver operating characteristic
snoRNAs: Small nucleolar RNAs
TNBC: Triple negative breast cancer
