# Supplementary material for: A plasma SNORD33 signature predicts platinum benefit in metastatic triple-negative breast cancer patients
Source: Mol Cancer. 2022 Jan 18;21:22. doi: 10.1186/s12943-022-01504-0 (PMC8764855; doi:10.1186/s12943-022-01504-0)
Supplement: Supplementary file 1 — Additional file 1. Methods. [file 12943_2022_1504_MOESM1_ESM.docx]

**METHODS**

**Patient enrollment**

This study was a retrospective cohort study. Peripheral blood specimens were obtained from 209 mTNBC patients who received platinum based chemotherapy as first-line treatment between years 2011 and 2019 from three clinical trials CBCSG006, NCT02546934 and NCT02341911 [1, 2]. mTNBC was defined as histologically confirmed unresectable recurrent or advanced breast cancer with ER<1%, PR<1% and HER2-negative. HER2-negative status was a score of 0 or 1 by immunohistochemistry or HER2/CEP17 ratio <2 by fluorescence in-situ hybridization analysis, according to the guideline of College of American Pathologists (CAP). No previous chemotherapy for metastatic disease before platinum-based chemotherapy. For patients receiving non-platinum regimen, peripheral blood specimens from 46 patients who received gemcitabine and paclitaxel as first-line treatment were collected from CBCSG006 study as control group [1]. Detailed clinical data were summarized in Supplementary Table 1. For lung adenocarcinoma patients, peripheral blood specimens were collected from 50 patients receiving platinum based chemotherapy as first-line treatment between years Dec 2019 and Nov 2020. Specimen were randomly sampled from eligible patients in the biobank of the Fudan University Shanghai Cancer Center (FUSCC). Eligible patients had stage IIIB or IV lung adenocarcinoma without previous chemotherapy. Patients with genomic alterations or previously treated with targeted therapy were permitted in this study. Detailed clinical data were summarized in Supplementary Table 4. Plasma was obtained 5 days before the first-line treatment initiation. All patients provided written informed consent for the use of their biological samples and clinical data for research purposes. The collection of blood samples and access to clinical data for research were approved by the Institutional Review Board of FUSCC.

**Clinical trial group**

We randomly assign 81 out of the 209 patients (combined three dataset of CBCSG006, NCT02546934 and NCT02341911) to the training cohort, leaving the remaining 128 patients to the validation cohort. We then merged the training and validation cohort for a combined analysis of PFS and OS. Patients from three clinical trials received the platinum-based chemotherapy as follows: For CBCSG006, patients received cisplatin plus gemcitabine (GP) (cisplatin 75 mg/m^2^ d1 and gemcitabine 1250 mg/m^2^ on d1, 8, q3w); for NCT02341911, patients received GP, same dose with CBCSG006 study, or carboplatin plus gemcitabine (GC) (carboplatin AUC=2 on day 1, 8 and gemcitabine 1000 mg/m^2^ on days 1, 8 q3w); for NCT02546934, patients received GP or cisplatin plus nab-paclitaxel (AP) (cisplatin 75 mg/m^2^ on d1 and nab-paclitaxel 125 mg/m^2^ on d1, 8 q3w).

Lung adenocarcinoma patients received platinum-based regimens including pemetrexed plus cisplatin (cisplatin 25 mg/m^2^ d1-3 and pemetrexed 500 mg/m^2^ on d1, q3w), pemetrexed plus carboplatin (carboplatin AUC=5 on d1 and pemetrexed 500 mg/m^2^ on d1, q3w), pemetrexed plus carboplatin plus bevacizumab (carboplatin AUC=5 on d1, pemetrexed 500 mg/m^2^ on d1 and bevacizumab 7.5mg/kg on d1, q3w), pemetrexed plus carboplatin plus pembrolizumab (carboplatin AUC=5 on d1, pemetrexed 500 mg/m^2^ on d1 and pembrolizumab 200 mg on d1, q3w) or paclitaxel plus carboplatin plus pembrolizumab (carboplatin AUC=2 on d1, 8, paclitaxel 80 mg/m^2^ on d1, 8 and pembrolizumab 200 mg on d1, q3w). Progression-free survival was defined as the time from the date of randomization to progression or death from any cause, whichever occurred first. Overall survival defined as the time from randomization to death from any cause. Tumor responses were confirmed by researchers according to Response Evaluation Criteria in Solid Tumors (RECIST) 1.1 criteria. Complete Response (CR): Disappearance of all target lesions. Any pathological lymph nodes (whether target or non-target) must have reduction in short axis to <10 mm. Partial Response (PR): At least a 30% decrease in the sum of diameters of target lesions, taking as reference the baseline sum diameters. Progressive Disease (PD): At least a 20% increase in the sum of diameters of target lesions, taking as reference the smallest sum on study (this includes the baseline sum if that is the smallest on study). In addition to the relative increase of 20%, the sum must also demonstrate an absolute increase of at least 5 mm. (Note: the appearance of one or more new lesions is also considered progression). Stable Disease (SD): Neither sufficient shrinkage to qualify for PR nor sufficient increase to qualify for PD, taking as reference the smallest sum diameters while on study.

**Plasma sample collection and processing**

We collected blood samples from mTNBC patients before first-line chemotherapy into vacuum blood tubes with anticoagulant, labeled the tube, and gently inverted the tube 8-10 times and then centrifuged the tube at 1500 × g for 10 min in a refrigerated centrifuge. We transferred the liquid component (plasma) into an RNase-free cryovial tube with transfer pipette and labeled the tube carefully. Plasma was frozen at −80 °C for next step experiments. Samples were maintained on ice while handling.

**RNA extraction from plasma**

snoRNAs from plasma was extracted using the miRNeasy Mini Kit (Qiagen, Valencia, CA, USA). Briefly, plasma was thawed on ice and high centrifuged at 100,000 rpm for 15 min to remove cellular debris completely. Next, 200 μl supernatant was lysed with 5 volumes of QIAzol solution. Small RNAs were enriched and purified according to the manufacturer’s protocol, with the exception that the enriched small RNAs were eluted in 20 μl RNase-free water.

**Quantification of SNORD33 expression by real-time RT-qPCR**

Expressions of the identified snoRNAs were tested in plasma and cells using real-time SYBR green RT-qPCR assay. For snoRNA-based RT-PCR assays, 4 μl of enriched small RNAs from plasma or 25 ng small RNAs from TNBC cell lines were reverse transcribed using the Tiangen MicroRNA Reverse Transcription Kit in a total reaction volume of 10 μl, according to the manufacturer’s instructions. RT products were diluted 1:9 and used as PCR templates. RT-qPCR was performed using TaqMan Universal PCR Master Mix (Applied Biosystems) according to the manufacturer’s protocol with the commercial U6 primer and the snoRNA-specific primers. The primer sequences were as follows. SNORD33 forward primer: 5'-GAACTTCTCCCACTCACATTCG-3',

SNORD33 reverse primer: 5'-GTGGCCTCAGATGGTAGTGC-3'; U6 forward primer: 5'-CTCGCTTCGGCAGCACA-3',

U6 reverse primer: 5'-AACGCTTCACGAATTTGCGT-3'. The Step One Plus Real Time PCR System (Applied Biosystems) was applied with the following cycling conditions: 95 °C for 10 min, 40 cycles at 95 °C for 15 sec, and 60 °C for 1 min. All assays were performed in triplicates, and one no-template control and two interplate controls were carried along in each experiment.

The average expression levels of snoRNAs in plasma or cells were normalized against U6 using the 2-ΔCt method. Differences between the groups were presented as ΔCt, indicating the difference between snoRNAs and Ct value of the normalizer U6. U6 RNA was proven as an internal control for ncRNA quantification [3, 4]. To ensure consistent measurements throughout all assays, for each PCR amplification reaction, one of the RNA samples was loaded as internal control to account for any plate-to-plate variation, and the results from each plate were normalized against internal normalization controls.

**BRCA mutation detection**

We used blood samples from 114 patients in which the homologous recombination genes including BRCA1 and BRCA2 were confirmed by targeted next-generation sequencing (NGS) for a comprehensive analysis of 1 ug genomic DNA samples were obtained from peripheral blood leukocytes. A synthesized 5'-biotinylated 120 bp DNA oligonucleotides bait covers BRCA1/2 [5]. A total of 188 amplicons with a mean amplicon target length of 196 bp (range 120 to 278 bp), were amplified to create sequencing libraries of the complete BRCA1/2 genes in a single tube, using PCR. Libraries were sequenced in next generation sequencing platform illumina Nextseq 500 using 75-bp paired-end reads. Each sample was sequenced at depth > 200x. Sequence data were mapped to the human genome (hg19) using Burrows-Wheeler Aligner (BWA) v0.7.12. Clinical significance of variants was annotated by ClinVar database (http://www.ncbi.nlm.nih.gov/clinvar), and classified into benign, likely-benign, variants of uncertain significance, likely-pathogenic, and pathogenic. Pathogenic and likely-pathogenic variants were considered as clinical pathogenic, while “likely benign” and “benign” were considered as clinical non-pathogenic, and excluded from further analysis.

**Cell culture**

Human TNBC cell line MDA-MB-231, MDA-MB-468 and SUM149PT, lung adenocarcinoma cell lines A549, PC9, NCI-H1650 and H4006, and bladder carcinoma cell lines TCCSUP and J82, were obtained from the American Type Culture Collection (ATCC). MDA-MB-231 and MDA-MB-468 were maintained in Leibovitz’s L15 medium (Life, USA) supplemented with 10% fetal bovine plasma (Life, USA) at 37°C in a humidified incubator without CO_2_. SUM149PT were maintained in Ham's F12 medium (Sigma-Aldrich) containing 5% fetal bovine plasma (Life, USA), 10 mM HEPES (Sigma-Aldrich), 5 μg/ml insulin (Sigma-Aldrich), and 1 μg/ml dexamethasone (Sigma-Aldrich). A549, PC9, NCI-H1650 and H4006 were maintained in RPMI-1640 medium (Gibco, USA) supplemented with 10% fetal bovine serum (Gibco, USA) at 37°C in a humidified incubator with 5% CO_2_. TCCSUP and J82 were maintained in MEM medium (BasalMedia, China) containing 10% fetal bovine serum (Gibco, USA), 1% Non Essential Amino Acids (Sigma-Aldrich), 1 mM Sodium Pyruvate (Sigma-Aldrich) at 37°C in a humidified incubator with 5% CO_2_. According to ATCC, BRCA of MDA-MB-231 and MDA-MB-468 are wide type, while BRCA of SUM149PT is germline mutated. All cell lines have been authenticated by STR and have recently been tested mycoplasma contamination negative.

**Generation of cisplatin-resistant MDA-MB-231 cell line**

TNBC cell lines, MDA-MB-231, were sub-sequently stably selected for cisplatin (DDP) resistance by growth in progressively increasing concentrations of cisplatin from 3 μM, 6 μM, 12 μM to 24 μM over 15 passages [6-8]. The cisplatin resistant subline was designated as MDA-MB-231/DDP, and were compared with the parental MDA-MB-231 cell line. Cell lines were grown in complete L15 containing 2 mmol/L L-glutamine, 1% nonessential amino acids, 1% penicillin-streptomycin, and 10% fetal bovine plasma at 37 °C. To maintain the resistant phenotype, the cisplatin-resistant cell lines were cultured in the presence of 20 μM cisplatin.

**RNA sequencing**

MDA-MB-231/DDP and parental MDA-MB-231 cells were sequenced at total RNA level. Total RNA was extracted using Trizol reagent kit (Invitrogen, Carlsbad, CA, USA) according to the manufacturer’s protocol. RNA quality was assessed on an Agilent 2100 Bioanalyzer (Agilent Technologies, Palo Alto, CA, USA) and checked using RNase free agarose gel electrophoresis. Strand-specific rRNA depleted RNA-seq library was constructed using VAHTS Total RNA-seq (H/M/R) Library Prep Kit (Vazyme, Nanjing, China). Briefly, rRNAs were removed and the retained RNAs were fragmented into short fragments by using fragmentation buffer and reverse transcribed into cDNA with random primers. Second-strand cDNA were synthesized by DNA polymerase I, RNase H, dNTP (dUTP instead of dTTP) and buffer. Next, the cDNA fragments were purified with VAHTSTM DNA Clean Beads, end repaired, poly(A) added, and ligated to Illumina sequencing adapters. Then UNG (Uracil-N-Glycosylase) was used to digest the second-strand cDNA. The digested products were purified with VAHTSTM DNA Clean Beads, PCR amplified to complete library construction. Libraries were sequenced using Illumina X10. Raw reads were filtered by fastp (version 0.18.0) to obtain high quality clean reads. Bowtie2 (version 2.2.8) was used for mapping reads to ribosome RNA (rRNA) database [9]. The rRNA mapped reads were then removed. The remaining reads were further used in RNA analysis including mRNA, lncRNA, miRNA, snoRNA, *et al*.

**Transfection of antisense oligonucleotides**

Cells were seeded in 6-well plates at 70% confluence. After 16 h, cells were transfected with the indicated antisense oligonucleotides (ASOs) using Lipofectamine 3000 Reagent (Thermo Fischer Scientific, Waltham, mA) for the following analyses. ASOs were purchased from RiboBio and the target sequences were as follows. ASO1: 5'-ACATTCGAGTTTCCCGACCA-3’ (lnc6180625014841); ASO2: 5'-GATGACTCCACATGCACTAC-3’ (lnc6180625014853); ASO3: 5'-GCACTACCATCTGAGGCCAC-3’ (lnc6180625014904)

**Flow cytometric analysis**

Cells were grown in 6-well plates to 80% confluence and treated with indicated concentration cisplatin for 48 h. After incubation with Annexin V-FITC and 7-AAD (BD Bioscience, San Jose, CA) according to the manufacturer’s recommendations, cell apoptosis was then analyzed using flow cytometry (FACScan; BD Biosciences) equipped with a Cell Quest software (BD Biosciences).

**Western blot**

Total protein was extracted from breast cancer cells in RIPA lysis buffer and separated in 10% or 12% sodium dodecyl sulfate-polyacrylamide gel electrophoresis (SDS-PAGE) before electro-transferred onto polyvinylidene difluoride (PVDF) membrane. The membrane was incubated with primary antibody overnight at 4 °C. Subsequently, the membrane was incubated with secondary antibody for 1 h at room temperature. Immunoreactive bands on the membrane were visualized by the enhanced chemiluminescence assay. The primary antibodies to β-actin (#3700, 1:3000, Cell signaling), cleaved-caspase 3 (#9661T, 1:1000, Cell signaling), cleaved-caspase 9 (#9505, 1:1000, Cell signaling), Mcl-1 (sc-74437, 1:1000, Santa Cruz Biotechnology) and Bcl-2 (sc-72235, 1:1000, Santa Cruz Biotechnology), MeCP2 (#3456, 1:1000, Cell signaling) were commercially obtained. The secondary monoclonal antibodies (115-035-146, 111-035-144, 1:10000) were purchased from Jackson Immunoresearch (USA).

**RNA pull-down assays**

RNA pull-down assays were performed as previously described [10]. Briefly, probes of sense and antisense SNORD33 were transcribed and labeled with a Biotin RNA Labeling Mix (Roche, IN, USA), followed by treatment with RNasefree DNase I (TaKaRa, Tokyo, Japan) and purification with a RNeasy Mini kit (Qiagen, Hilden, Germany). MDA-MB-231/DDP and parental MDA-MB-231 cells were lysed in RIPA buffer supplemented with RNase and proteinase inhibitors, and then were incubated with equal moles of each biotinylated RNA at 4 °C for 4 h. The mixture was added to 40 μl of prewashed streptavidin beads (Thermo Fisher Scientific, IL, USA) and was incubated on a rotator at room temperature for 1 h. The beads were washed briefly five times with NT2 buffer and boiled in SDS loading buffer. The protein samples were separated by SDS-PAGE. Differential band between sense and anti-sense were collected and subjected to mass spectrometry and retrieved in human proteomic library.

**RNA immunoprecipitation**

RNA immunoprecipitation (RIP) experiments were performed using a Magna RIP RNA-Binding Protein Immunoprecipitation Kit (Millipore) according to the manufacturer's instructions [10]. Briefly, MDA-MB-231 cells were lysed in RIP lysis buffer. Magnetic beads were pre-incubated with MeCP2 antibodies (#3456, 1:25, Cell Signaling Technology) for 30 min at room temperature. The cell lysates were immunoprecipitated with the above beads for 6 h at 4 °C. Then, RNA was purified and detected by qRT-PCR.

**Chromatin immunoprecipitation (ChIP) assay**

ChIP analysis was performed using Chromatin Immunoprecipitation (ChIP) Assay Kit (Merck) [11]. Briefly, MDA-MB-231 were cross-linked using 1% formaldehyde solution for 10 min at room temperature and quenched with 125 mM glycine. DNA ranging from 200 to 800 bp were sheared by ultrasonication. The lysate of sheared chromatin from 1×10^7^ cells was incubated with anti-MeCP2 antibody (#10861-1-AP, 4 ug for 1×10^7^ cells, Proteintech) or IgG in ChIP buffer. DNA fragments were purified from immunoprecipitants. Immunoprecipitated DNAs were analyzed by qRT-PCR. The ChIP primers are listed below:

| GADD45A-F | TTGGGTTGTTAGGGATTTTTATATG |
| --- | --- |
| GADD45A-R | AAAATCTTTTCCACAAAAAACAAAA |
| FOXF1-F | AGGTGGTGCCTCCCCCCAAAAAATA |
| FOXF1-R | CTCTCTCCTCCCTCTTTCTTTCTCG |
| MYOD1-F | CTGCCGCCCCCAGCGAGGGAGAGAG |
| MYOD1-R | AGAGCACCTGGTATATCGGG |
| CLDN6-F | CCCATCCCCACGCACGCTT |
| CLDN6-R | GCCACCTGGATGGGCGAGTC |
| CDKL5-F | AATCCTCGGGCTGACAGTTCTT |
| CDKL5-R | ACGAAGGATGGCTTATCTCGGT |

**Co-Immunoprecipitation assay**

Co-Immunoprecipitation assay was performed as described in the previous study [12]. Briefly, MDA-MB-231 cells were harvested and lysed in immunoprecipitation lysis buffer. Supernatants were incubated with anti-MeCP2 antibody (#3456, 1:25, Cell signaling) at 4 °C overnight on a rotator, followed by addition of 40 μl prewashed protein A/G agarose beads. The bound proteins were eluted by elution buffer, boiled in loading buffer, and then detected by western blot using HDAC1 (#sc-81598, 1:1000, Santa Cruz) or mSIN3A (#sc-5299, 1:1000, Santa Cruz) antibody.

**Cell viability assay**

Cells were seeded in 96-well plates at a density of 5000 cells in 100 μl medium per well. After 24 h, cells were treated with indicated concentrations of cisplatin for 48 h, and the cell viability was assessed by CCK-8 assay (Beyotime Biotechnology) according to the manufacturer’s instructions. The optical density values were measured at 450 nm.

**Colony formation assay**

For colony formation assay, 500 cells were seeded per well in a six-well plate. The cells were incubated with cisplatin-containing medium for about 2 weeks at 37 °C, and then fixed with 4% paraformaldehyde and stained with crystal violet (Beyotime, China).

**Statistical analysis**

Statistical analyses were performed using GraphPad Prism version 7 and SPSS.22. The Student’s t-test was used to analyze the differential RNA expression detected by RNA sequence. Chi-square test or Fisher’s exact test was occupied for categorical variables. Cox regression was employed to determine prognostic performance. Kaplan–Meier method were used to estimate PFS and OS. The multiple Cox proportional hazards model was performed to assess risk factors for PFS. We proposed visual nomogram to predict the PFS probability. The nomogram is composed of graphical lines of points, risk factors, total points, and probability of event. The length of each risk factor’s line reflected the regression coefficient estimated by multiple Cox proportional hazards model. The point system works by ranking the regression coefficients, with SNORD33 expression converted into 100 points, followed by number of metastatic sites and liver metastasis (yes/no). The prediction performance of the nomogram was assessed by the discrimination and calibration [13]. A concordance index (C index) was calculated to estimate the discriminative ability of the nomogram. A C index larger than 0.5 indicates prediction performance better than random guessing, with 1 indicating perfect discrimination [14]. A visual calibration curve was plotted to evaluate the consistency between the nomogram-predicted probabilities and observed probabilities [15]. A perfect nomogram prediction may obtain a 45-degree line. In addition, the 1000 bootstrap resamples for internal validation were utilized to assess the predictive accuracies of this nomogram. All statistical tests were two-sided and *P*<0.05 was considered statistically significant. All analyses were performed in *R* software (version 4.0.3; The R Foundation for Statistical Computing, Vienna, Austria), with the “*rms*” package for the nomogram.

**REFERENCES**

1. Hu XC, Zhang J, Xu BH, Cai L, Ragaz J, Wang ZH, et al. Cisplatin plus gemcitabine versus paclitaxel plus gemcitabine as first-line therapy for metastatic triple-negative breast cancer (CBCSG006): a randomised, open-label, multicentre, phase 3 trial. Lancet Oncol. 2015; 16(4): 436-46.

2. Xie Y, Gu B, Hu X, Zhang Y, Zhang J, Wang Z, et al. Heterogeneity of targeted lung lesion predicts platinum-based first-line therapy outcomes and overall survival for metastatic triple-negative breast cancer patients with lung metastasis: a "PET biopsy" method. Cancer Manag Res. 2019; 11: 6019-27.

3. Xing L, Todd NW, Yu L, Fang H, and Jiang F. Early detection of squamous cell lung cancer in sputum by a panel of microRNA markers. Mod Pathol. 2010; 23(8): 1157-64.

4. Park NJ, Zhou H, Elashoff D, Henson BS, Kastratovic DA, Abemayor E, et al. Salivary microRNA: discovery, characterization, and clinical utility for oral cancer detection. Clin Cancer Res. 2009; 15(17): 5473-7.

5. Zhang J, Lin Y, Sun XJ, Wang BY, Wang ZH, Luo JF, et al. Biomarker assessment of the CBCSG006 trial: a randomized phase III trial of cisplatin plus gemcitabine compared with paclitaxel plus gemcitabine as first-line therapy for patients with metastatic triple-negative breast cancer. Ann Oncol. 2018; 29(8): 1741-7.

6. Bauer JA, Trask DK, Kumar B, Los G, Castro J, Lee JS, et al. Reversal of cisplatin resistance with a BH3 mimetic, (-)-gossypol, in head and neck cancer cells: role of wild-type p53 and Bcl-xL. Mol Cancer Ther. 2005; 4(7): 1096-104.

7. Lukyanova NY, Rusetskya NV, Tregubova NA, and Chekhun VF. Molecular profile and cell cycle in MCF-7 cells resistant to cisplatin and doxorubicin. Exp Oncol. 2009; 31(2): 87-91.

8. Wu Y, Zhou Y, He J, Sun H, and Jin Z. Long non-coding RNA H19 mediates ovarian cancer cell cisplatin-resistance and migration during EMT. Int J Clin Exp Pathol. 2019; 12(7): 2506-15.

9. Langdon WB. Performance of genetic programming optimised Bowtie2 on genome comparison and analytic testing (GCAT) benchmarks. BioData Min. 2015; 8(1): 1.

10. Xiu B, Chi Y, Liu L, Chi W, Zhang Q, Chen J, et al. LINC02273 drives breast cancer metastasis by epigenetically increasing AGR2 transcription. Mol Cancer. 2019; 18(1): 187.

11. Zhao Y, Yao D, Li Y, Zhang S, Tao Z, Zhang L, et al. Loss of polarity protein Par3 is mediated by transcription factor Sp1 in breast cancer. Biochem Biophys Res Commun. 2021; 561: 172-9.

12. Zhao GX, Xu YY, Weng SQ, Zhang S, Chen Y, Shen XZ, et al. CAPS1 promotes colorectal cancer metastasis via Snail mediated epithelial mesenchymal transformation. Oncogene. 2019; 38(23): 4574-89.

13. Iasonos A, Schrag D, Raj GV, and Panageas KS. How to build and interpret a nomogram for cancer prognosis. J Clin Oncol. 2008; 26(8): 1364-70.

14. Bandos AI, Rockette HE, Song T, and Gur D. Area under the free-response ROC curve (FROC) and a related summary index. Biometrics. 2009; 65(1): 247-56.

15. Harrell FE, Jr., Lee KL, and Mark DB. Multivariable prognostic models: issues in developing models, evaluating assumptions and adequacy, and measuring and reducing errors. Stat Med. 1996; 15(4): 361-87.
