# Supplementary material for: A plasma SNORD33 signature predicts platinum benefit in metastatic triple-negative breast cancer patients
Source: Mol Cancer. 2022 Jan 18;21:22. doi: 10.1186/s12943-022-01504-0 (PMC8764855; doi:10.1186/s12943-022-01504-0)
Supplement: Supplementary file 2 — Additional file 2: Supplementary Figure 1. Aberrantly expressing RNA in 231/DDP cells. Supplementary Figure 2. SNORD33 knockdown increases proliferation and decreases apoptosis of TNBC cells. Supplementary Figure 3. Reduced SNORD33 level is correlated with poor prognosis of mTNBC patients who received first-line platinum-based chemotherapy. Supplementary Figure 4. Increased cell viability was observed in cisplatin treated cell lines with SNORD33 knockdown. Supplementary Figure 5. Reduced SNORD33 level correlates with poor prognosis of non-small cell lung cancer (NSCLC) patients who received first-line platinum-based chemotherapy. Supplementary Figure 6. MeCP2 is a candidate protein binding with SNORD33. Supplementary Figure 7. Down-regulation of MeCP2 partially rescues SNORD33 knockdown increased cell colony formation. Supplementary Figure 8. Down-regulation of MeCP2 rescues SNORD33 knockdown decreased cell apoptosis and induced alteration of apoptotic markers. [file 12943_2022_1504_MOESM2_ESM.docx]

**Supplementary Figure 1** Wang *et al.*


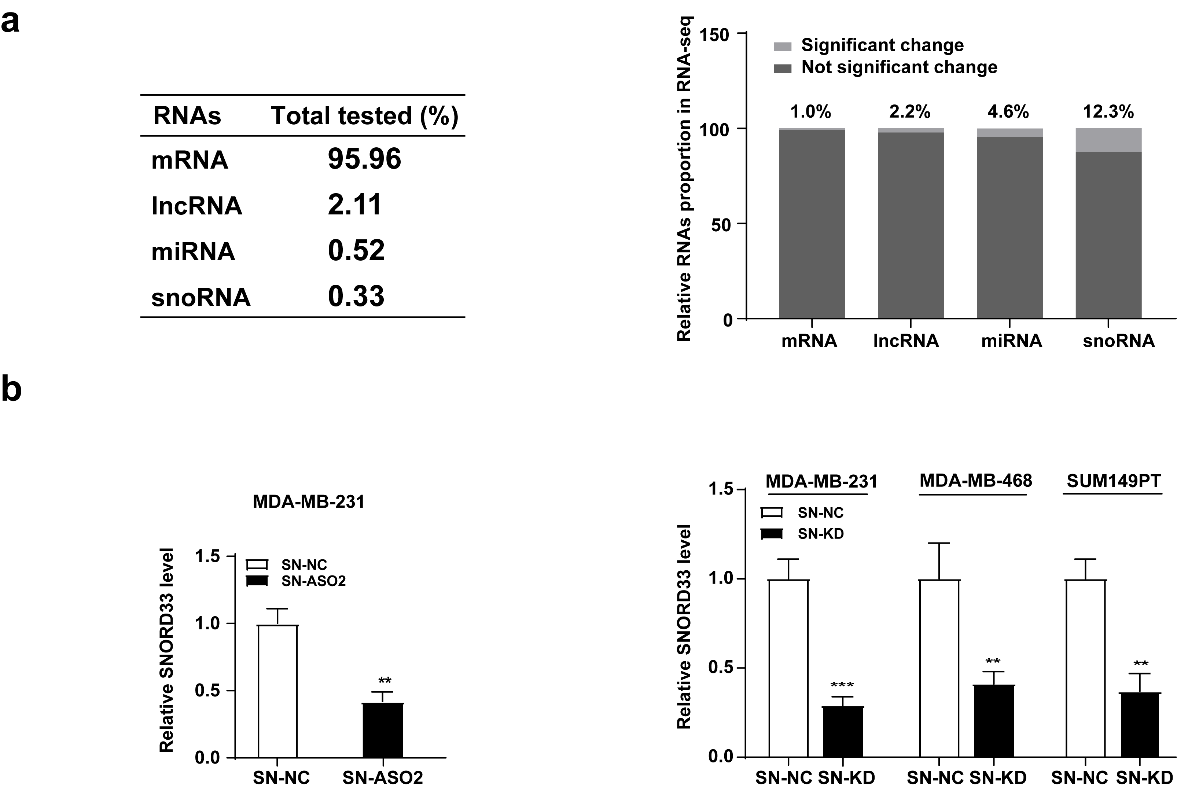


**Supplementary Figure 1 | Aberrantly expressing RNA in 231/DDP cells.**

**a** Significantly changed RNA percentage in total detected RNAs was measured using RNA-seq analyses of 231 and 231/DDP cells (left panel). The small nucleolar RNAs (snoRNAs) showed the highest variation rate (12.3%), compared with other groups, such as mRNA (1.0%), lncRNAs (2.2%) and miRNAs (4.6%) (right panel); significantly changed RNA were selected by using threshold values of >2 and <-2-fold change and *P*<0.05; n=3; two-tailed t test. **b** SNORD33 expression was successfully silenced by antisense oligonucleotide 2 (ASO2). The antisense oligonucleotide were transfected into MDA-MB-231, as well as MDA-MB-468 and SUM149PT cells. qRT-PCR was performed to validate the expression of SNORD33. SN-NC, control; SN-KD, ASO2. n=3; ** represents *P*<0.01, *** represents *P*<0.001; two-tailed t test.

**Supplementary Figure 2** Wang *et al.*


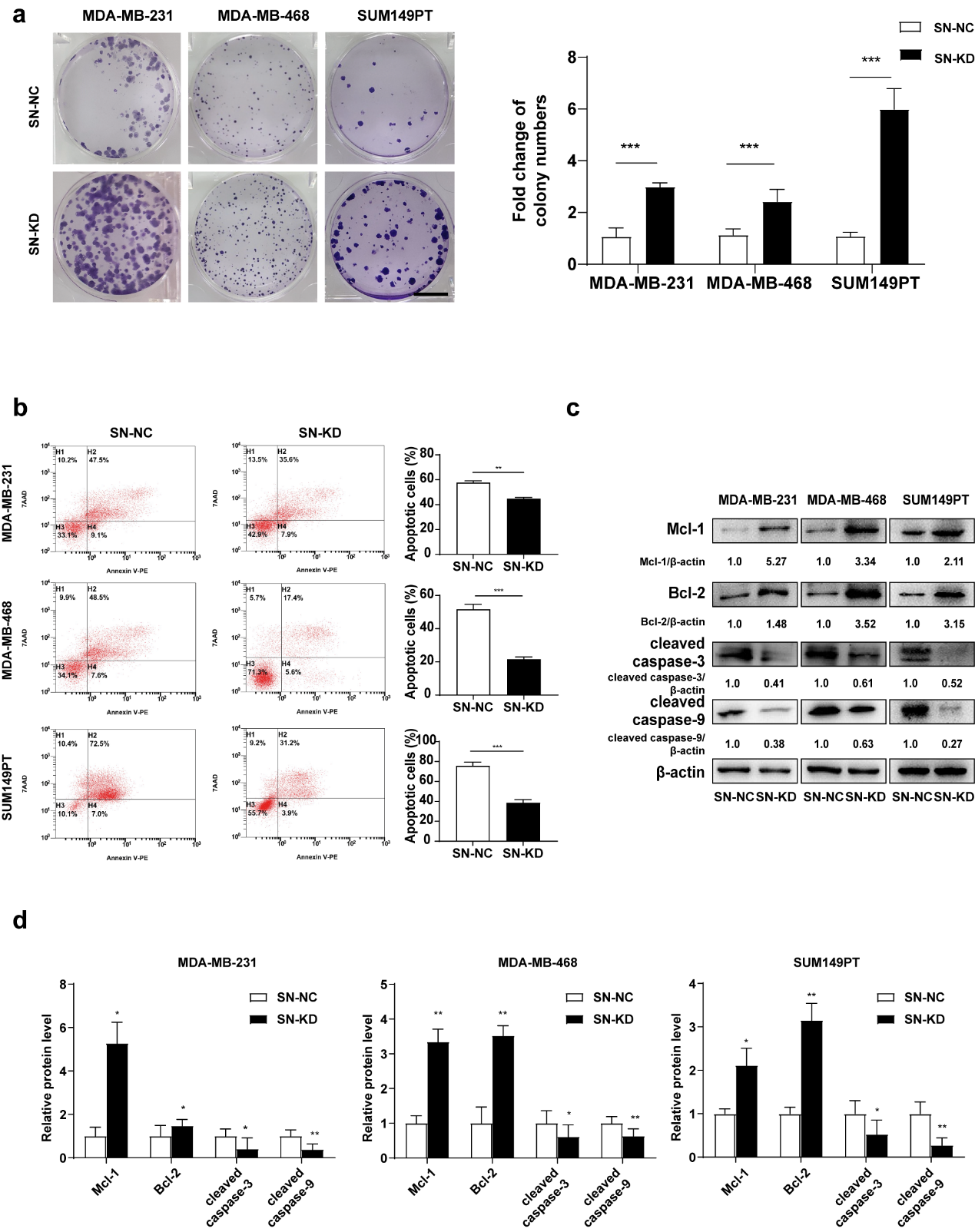


**Supplementary Figure 2 |** **SNORD33 knockdown increases proliferation and decreases apoptosis of TNBC cells.**

**a** SNORD33 knockdown increases cell colony formation of cisplatin treated TNBC cells. n=3; *** represents *P*<0.001; two-tailed t test. **b** SNORD33 knockdown decreases cisplatin induced apoptosis of TNBC cells. MDA-MB-231, MDA-MB-468 and SUM149PT cells were transfected with SNORD-ASO2, followed by treating with cisplatin for 48 h. Cell apoptosis was measured by Annexin V and 7-AAD staining. The percentages of cisplatin induced apoptotic cells were quantified and analyzed. n=3; ** represents *P*<0.01, *** represents *P*<0.001; two-tailed t test. **c** SNORD33 knockdown changes apoptosis associated protein in cisplatin induced TNBC cells. MDA-MB-231, MDA-MB-468 and SUM149PT cells with SNORD33 knockdown were treated as mentioned above and the cell lysates were then subjected to western blot analysis with the indicated antibodies. The concentration of cisplatin is 10 μM for MDA-MB-231, 8 μM for MDA-MB-468 and 3 μM for SUM-149PT. **d** The relative protein levels of **c** were normalized against β-actin. n=3; * represents *P*<0.05, ** represents *P*<0.01; two-tailed t test.

**Supplementary Figure 3** Wang *et al.*


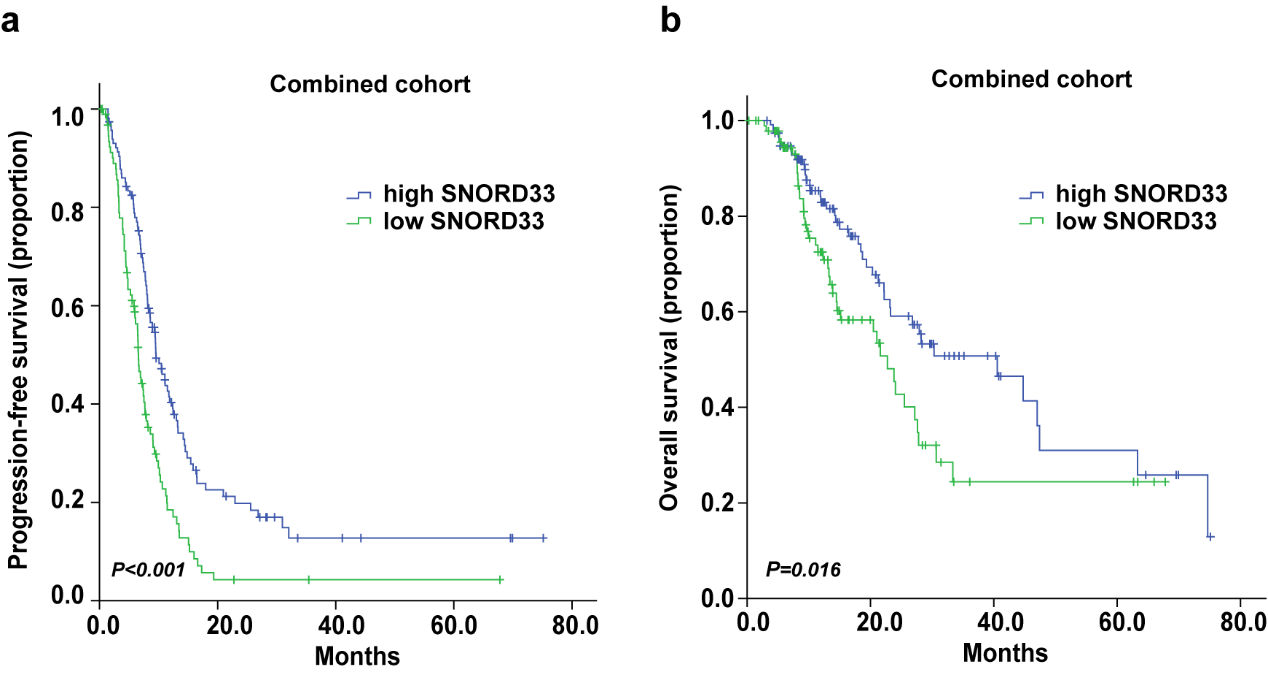


**Supplementary Figure 3 | Reduced SNORD33 level is correlated with poor prognosis of mTNBC patients who received first-line platinum-based chemotherapy.**

Kaplan-Meier survival curves for PFS **a**) and OS **b**) in combined cohorts (n=209). cut-off threshold was median value in training cohort; log-rank test. PFS, *P*<0.001; OS, *P*=0.016.

**Supplementary Figure 4** Wang *et al.*


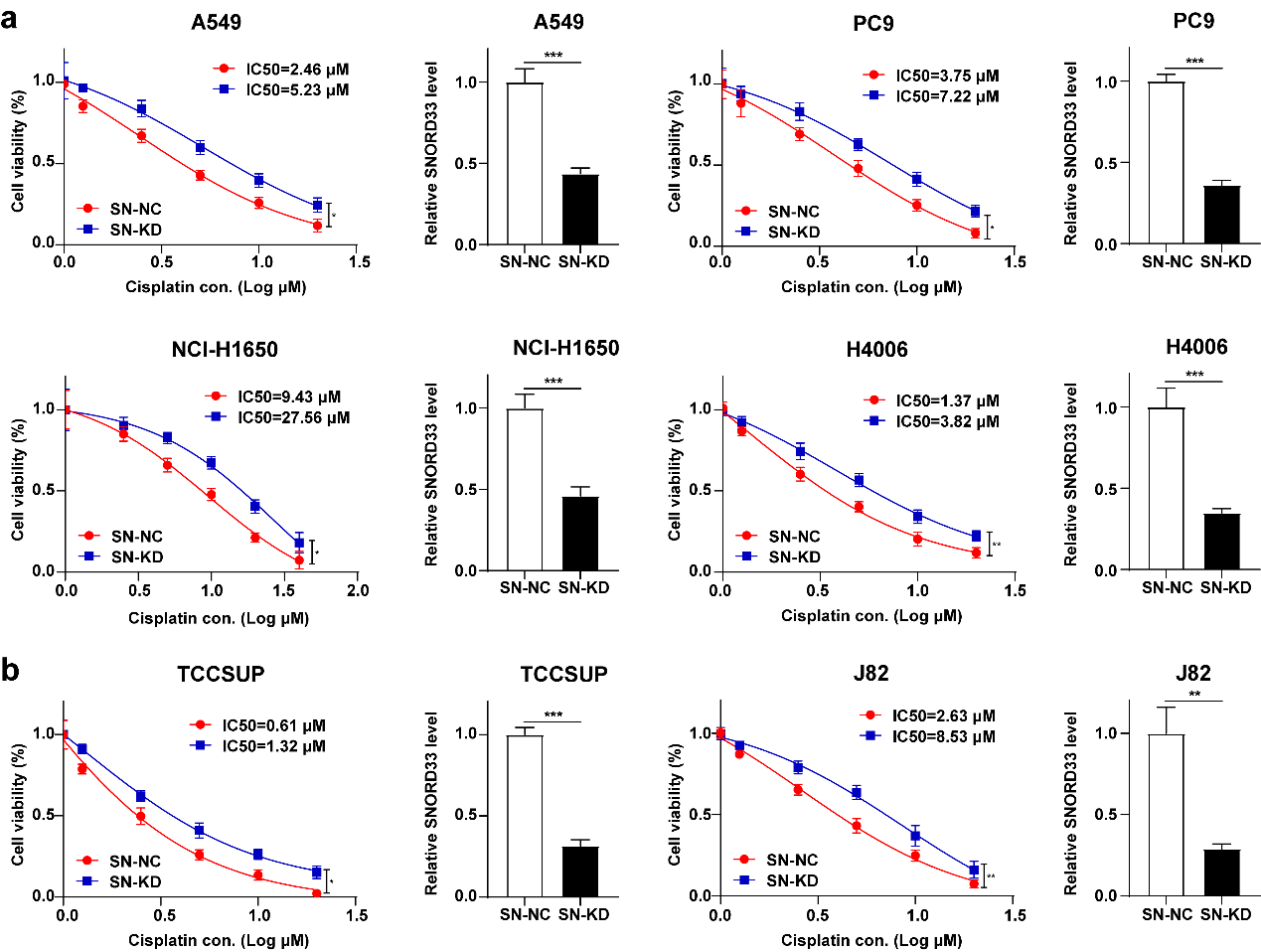


**Supplementary Figure 4 | Increased cell viability was observed in cisplatin treated cell lines with SNORD33 knockdown.**

SNORD33 was knocked down in lung adenocarcinoma A549, PC9, NCI-H1650, H4006 cell lines **a)**, and urinary bladder transitional cell carcinoma TCCSUP, J82 cell lines **b)**. Cells were then treated with cisplatin at indicated concentrations for 48 h and cell viability was determined by CCK8 assay. n=3; ** represents *P*<0.01, *** represents *P*<0.001; two-tailed t test.

**Supplementary Figure 5** Wang *et al.*


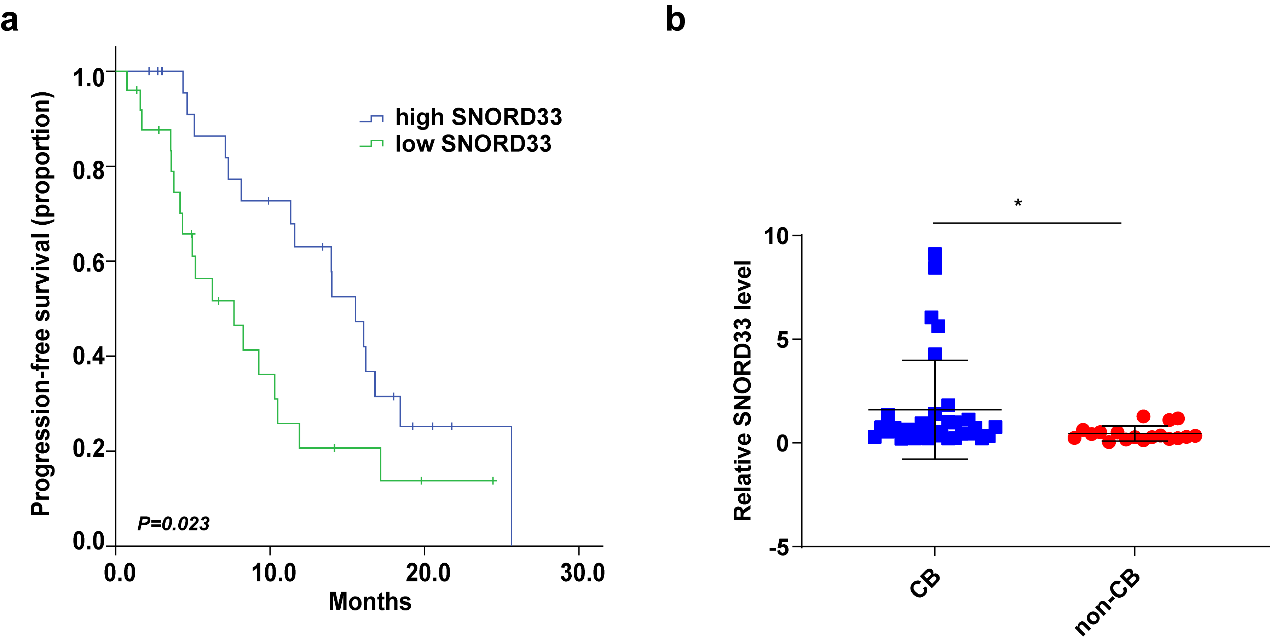


**Supplementary Figure 5 |** **Reduced SNORD33 level correlates with poor prognosis of non-small cell lung cancer (NSCLC) patients who received first-line platinum-based chemotherapy.**

**a** Kaplan-Meier survival curves for PFS (n=50). Cut-off threshold was median value; log-rank test. PFS, *P*=0.023. **b** Plasma SNORD33 level was significantly higher in patients reaching clinical benefit (CB) (CB, CR+PR+SD＞6 months). 1.36 versus 0.34, *P*=0.048; two-tailed t test.

**Supplementary Figure 6** Wang *et al.*


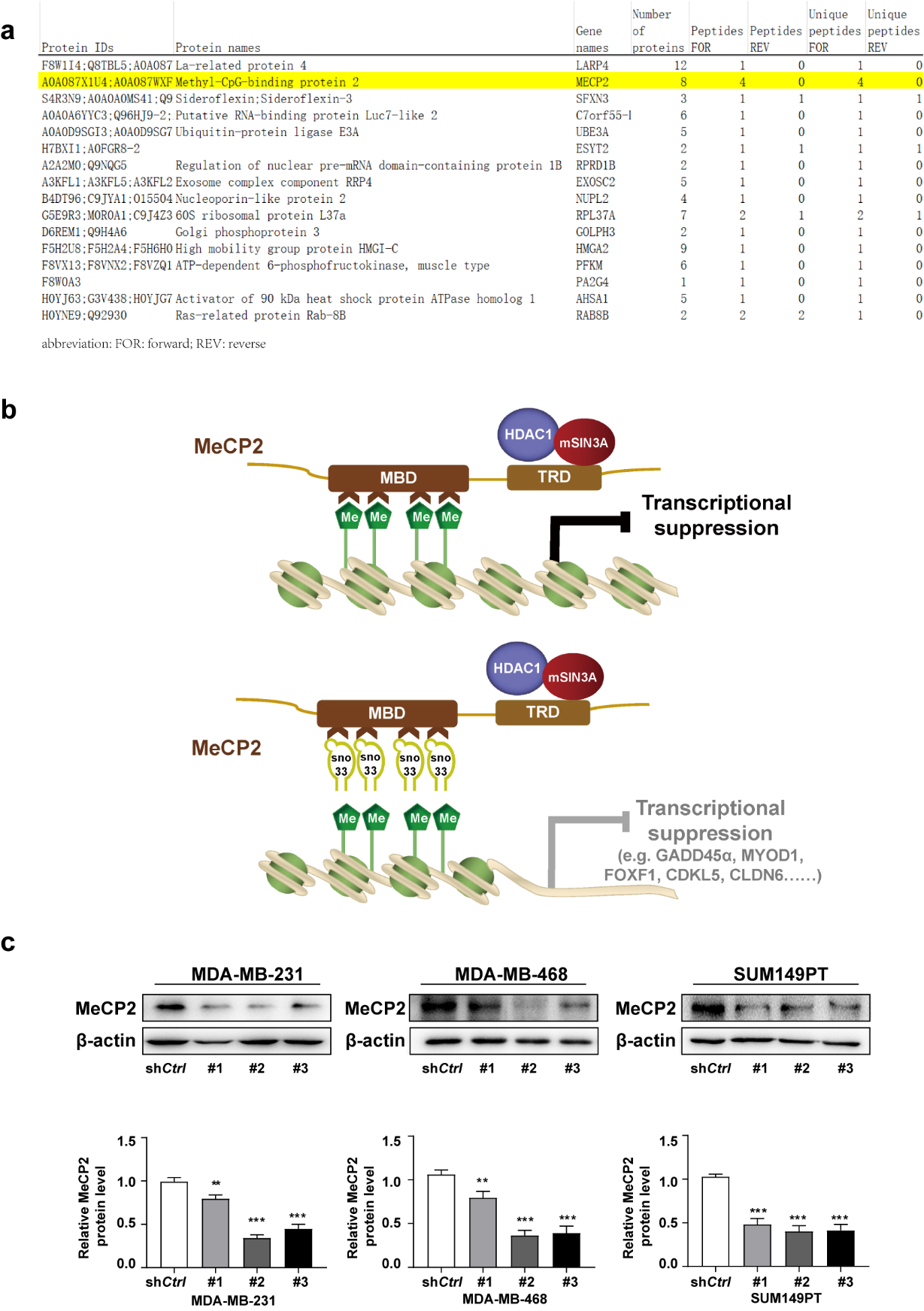


**Supplementary Figure 6 | MeCP2 is a candidate protein binding with SNORD33.**

**a** Mass spectrometry shows that MeCP2 is a candidate protein binding with SNORD33. **b** A graphic model illustrating the role of SNORD33 in regulating MeCP2 target genes. MeCP2 binds to CpG-methyl groups on DNA via its MBD domain, and/or interacts with its co-repressor mSIN3A and HDAC1 via its TRD to cause histone deacetylation, chromatin compaction, resulting in target-gene repression. SNORD33 reduces the binding of MeCP2 to target genes and relieves the repression of MeCP2 on its target genes. However, SNORD33 has little influence on the formation of MeCP2/ mSIN3A/HDAC1 complex. Me: methylated CpG, MBD: methyl-CpG binding domain, TRD: transcription repression domain. **c** The efficiency of *MeCP2* knockdown in MDA-MB-231, MDA-MB-468 and SUM149PT cells was detected by western blotting. n=3; ** represents *P*<0.01, *** represents *P*<0.001; two-tailed t test.

**Supplementary Figure 7** Wang *et al.*


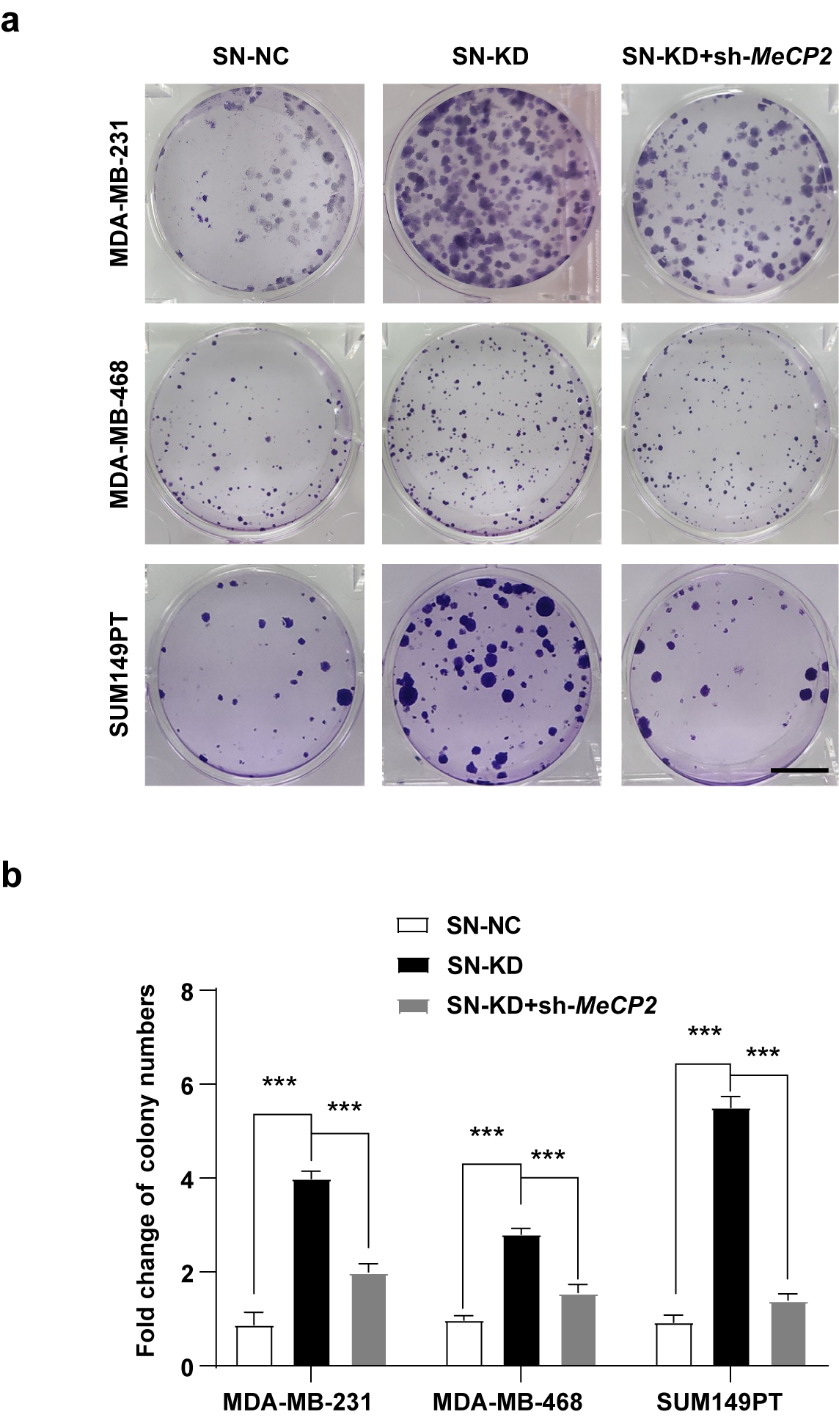


**Supplementary Figure 7 |** **Down-regulation of MeCP2 partially rescues SNORD33 knockdown increased cell colony formation.**

*MeCP2* was knocked down in SNORD33 knockdown MDA-MB-231, MDA-MB-468, SUM149PT cells. The colony formation was determined **a)** and normalized against control group **b)**. n=3; *** represents *P*<0.001; two-tailed t test.

**Supplementary Figure 8** Wang *et al.*


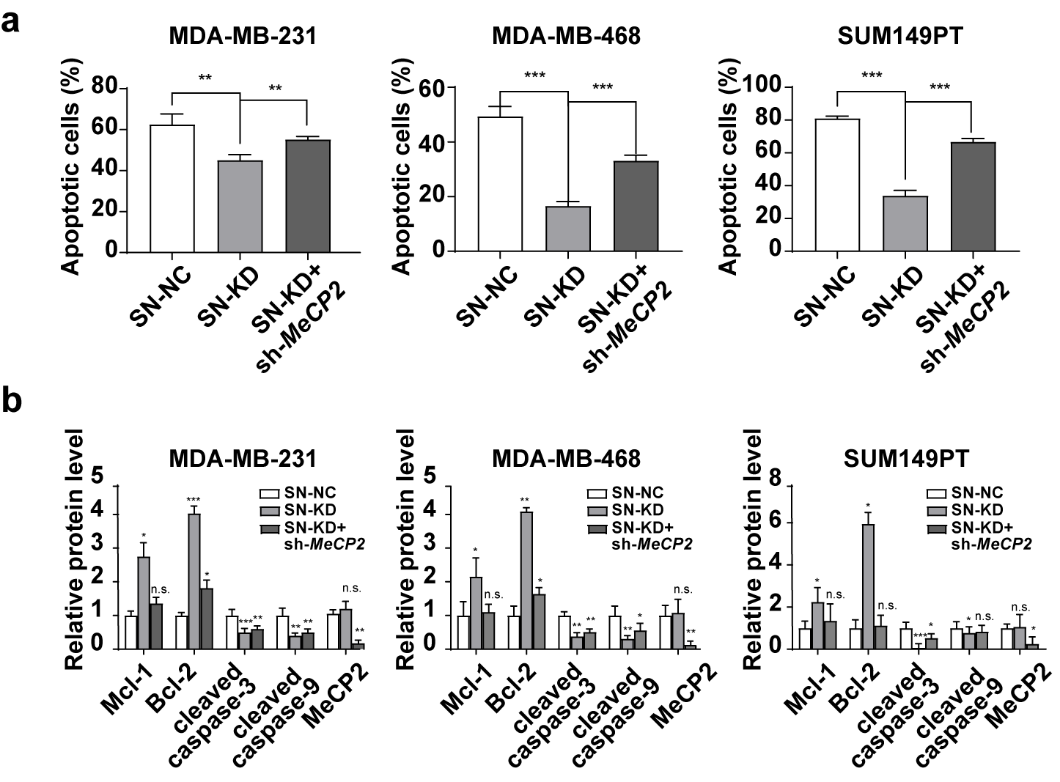


**Supplementary Figure 8 | Down-regulation of MeCP2 rescues SNORD33 knockdown decreased cell apoptosis and induced alteration of apoptotic markers.**

The relative protein levels of Fig. 2k and l were shown against β-actin in **a**) and **b**), respectively. n=3; n.s. represents *P*>0.05, * represents *P*<0.05, ** represents *P*<0.01, *** represents *P*<0.001; two-tailed t test.
