# Supplementary material for: A plasma SNORD33 signature predicts platinum benefit in metastatic triple-negative breast cancer patients
Source: Mol Cancer. 2022 Jan 18;21:22. doi: 10.1186/s12943-022-01504-0 (PMC8764855; doi:10.1186/s12943-022-01504-0)
Supplement: Supplementary file 3 — Additional file 3: Supplementary Table 1. Baseline characteristics in mTNBC patients received first-line platinum-containing regimens. Supplementary Table 2. Univariate and multivariate analysis of prognostic factors associated with progression-free survival and overall survival in the combined cohort. Supplementary Table 3. Baseline characteristics in mTNBC patients received first-line non-platinum-containing regimens. Supplementary Table 4. Baseline characteristics in lung adenocarcinoma patients received first-line platinum-containing regimens. Supplementary Table 5. Univariate and multivariate analysis of prognostic factors associated with progression-free survival in lung adenocarcinoma patients. [file 12943_2022_1504_MOESM3_ESM.docx]

**Supplementary Table 1.** Baseline characteristics in mTNBC patients received first-line platinum-containing regimens.

| **Clinicopathologic characteristics** | **SNORD33 expression** | | | | | | | | | | | |
| --- | --- | --- | --- | --- | --- | --- | --- | --- | --- | --- | --- | --- |
|  | **Training** | | | | | **Validation** | | | **Combined** | | | |
|  | **high** | | **low** | ***P*** | **high** | | **low** | ***P*** | | **high** | **low** | ***P*** |
| Age (years) |  | |  |  |  | |  |  | |  |  |  |
| <40 | 2 | | 7 | 0.146 | 14 | | 10 | 0.691 | | 16 | 17 | 0.562 |
| ≥40 | 39 | | 33 |  | 56 | | 48 |  | | 99 | 77 |  |
| Menopausal status |  | |  |  |  | |  |  | |  |  |  |
| Premenopausal | 9 | | 11 | 0.563 | 34 | | 32 | 0.457 | | 43 | 43 | 0.451 |
| Postmenopausal | 32 | | 29 |  | 36 | | 26 |  | | 72 | 51 |  |
| Disease free interval |  | |  |  |  | |  |  | |  |  |  |
| Primary | | 4 | 5 |  | 9 | | 11 |  | | 13 | 16 |  |
| <1 year | 3 | | 2 | 0.854 | 10 | | 8 | 0.636 | | 13 | 10 | 0.615 |
| ≥1 year | 34 | | 33 |  | 51 | | 39 |  | | 85 | 72 |  |
| Number of metastatic organs |  | |  |  |  | |  |  | |  |  |  |
| 1 | 19 | | 18 |  | 29 | | 16 |  | | 48 | 34 |  |
| 2 | 11 | | 12 | 0.948 | 18 | | 18 | 0.262 | | 30 | 29 | 0.448 |
| ≥3 | 11 | | 10 |  | 23 | | 24 |  | | 37 | 31 |  |
| Visceral metastasis |  | |  |  |  | |  |  | |  |  |  |
| Liver metastasis | 7 | | 7 | 0.959 | 17 | | 11 | 0.469 | | 24 | 18 | 0.558 |
| Lung metastasis | 17 | | 22 | 0.223 | 33 | | 30 | 0.606 | | 52 | 50 | 0.247 |

Pearson χ^2^ tests for all analyses.

**Supplementary Table 2**. Univariate and multivariate analysis of prognostic factors associated with progression-free survival and overall survival in the combined cohort.

| **Variables** | **Progression free survival** | | | | | **Overall survival** | | | | |
| --- | --- | --- | --- | --- | --- | --- | --- | --- | --- | --- |
|  | **Univariate** | **Multivariate** | | | **Univariate** | | **Multivariate** | | |  |
|  | ***P*** | **HR** | **95%CI** | ***P*** | ***P*** | | **HR** | **95%CI** | ***P*** | |
| Age (<40 years) | 0.334 |  |  | NA | 0.215 | |  |  | NA | |
| Menopausal status (premenopausal) | 0.247 |  |  | NA | 0.092 | |  |  | NA | |
| Disease free interval (<1 year) | 0.674 |  |  | NA | 0.116 | |  |  | NA | |
| Number of metastatic sites (≥3 site) | **<0.001*** | 0.531 | 0.356-0.792 | **0.002*** | **<0.001*** | | 0.297 | 0.166-0.532 | **<0.001*** | |
| Lung metastasis (yes) | 0.691 |  |  | NA | 0.340 | |  |  | NA | |
| Liver metastasis (yes) | **<0.001*** | 0.583 | 0.394-0.865 | **0.009*** | **0.016*** | | 0.733 | 0.435-1.236 | 0.244 | |
| SNORD33 (low expression) | **<0.001*** | 0.481 | 0.351-0.659 | **<0.001*** | **0.016*** | | 0.739 | 0.469-1.163 | 0.191 | |

Cox proportional hazards regression model was used in univariate analysis. Bold items were considered statistically significant. HR, hazard ratio; CI, confidence interval.

**Supplementary Table 3.** Baseline characteristics in mTNBC patients received first-line non-platinum-containing regimens.

| **Clinicopathologic characteristics** | **SNORD33 expression** | | |
| --- | --- | --- | --- |
|  | **high** | **low** | ***P* value** |
| Age (years) |  |  |  |
| <40 | 9 | 3 | 0.053 |
| ≥40 | 14 | 19 |  |
| Menopausal status |  |  |  |
| Premenopausal | 13 | 11 | 0.661 |
| Postmenopausal | 10 | 11 |  |
| Disease free interval |  |  |  |
| Primary | 3 | 1 |  |
| <1 year | 4 | 2 | 0.375 |
| ≥1 year | 16 | 19 |  |
| Number of metastatic organs |  |  |  |
| 1 | 6 | 6 |  |
| 2 | 7 | 8 | 0.875 |
| ≥3 | 10 | 8 |  |
| Visceral metastasis |  |  |  |
| Liver metastasis | 6 | 3 | 0.502 |
| Lung metastasis | 15 | 15 | 0.833 |

Pearson χ^2^ tests for all analyses.

**Supplementary Table 4.** Baseline characteristics in lung adenocarcinoma patients received first-line platinum-containing regimens.

| **Clinicopathologic characteristics** | **SNORD33 expression** | | |
| --- | --- | --- | --- |
|  | **high** | **low** | ***P* value** |
| Age (years) |  |  |  |
| <55 | 4 | 8 | 0.185 |
| ≥55 | 21 | 17 |  |
| Gender |  |  |  |
| Male | 20 | 17 | 0.33 |
| Female | 5 | 8 |  |
| Previous systemic neoadjuvant/adjuvant therapy |  |  |  |
| Yes | 1 | 2 | 1.00 |
| No | 24 | 23 |  |
| Number of metastatic organs |  |  |  |
| 1 | 8 | 4 |  |
| 2 | 10 | 8 | 0.19 |
| ≥3 | 7 | 13 |  |
| Visceral metastasis |  |  |  |
| Liver metastasis | 3 | 3 | 1.00 |
| Lung metastasis | 8 | 9 | 1.00 |
| Mutation |  |  |  |
| EGFR mutations | 5 | 6 | 0.73 |
| ALK rearrangements | 0 | 1 | 0.47 |
| ROS1 rearrangements | 1 | 1 | 1.00 |
| KRAS mutations | 5 | 1 | 0.19 |
| Previous treated for metastatic disease |  |  |  |
| Gefitinib | 1 | 2 | 1.00 |
| Afatinib | 0 | 1 | 1.00 |
| Osimertinib | 1 | 0 | 1.00 |
| Platinum-containing regimens |  |  | 0.17 |
| Pemetrexed+cisplatin | 0 | 4 |  |
| Pemetrexed+carboplatin | 4 | 5 |  |
| Pemetrexed+carboplatin+bevacizumab | 10 | 10 |  |
| Pemetrexed+carboplatin+pembrolizumab | 7 | 4 |  |
| Paclitaxel+carboplatin+pembrolizumab | 3 | 3 |  |

Pearson χ^2^ tests for all analyses.

**Supplementary Table 5**. Univariate and multivariate analysis of prognostic factors associated with progression-free survival in lung adenocarcinoma patients.

| **Variables** | **Progression free survival** | | | | |
| --- | --- | --- | --- | --- | --- |
|  | **Univariate** | **Multivariate** | | |  |
|  | ***P*** | **HR** | **95%CI** | ***P*** |  |
| Age (<60 years) | 0.91 |  |  | NA |  |
| Gender (Male) | 0.93 |  |  | NA |  |
| Number of metastatic sites (≥3 site) | **0.042*** | 2.10 | 1.02-4.18 | **0.042*** |  |
| Lung metastasis (yes) | 0.79 |  |  | NA |  |
| Liver metastasis (yes) | 0.94 |  |  | NA |  |
| EGFR mutations (yes) | 0.28 |  |  | NA |  |
| SNORD33 (low expression) | **0.026*** | 2.20 | 1.10-4.41 | **0.032*** |  |

Cox proportional hazards regression model was used in univariate analysis. Bold items were considered statistically significant. HR, hazard ratio; CI, confidence interval.
