# Supplementary material for: A plasma SNORD33 signature predicts platinum benefit in metastatic triple-negative breast cancer patients
Source: Mol Cancer. 2022 Jan 18;21:22. doi: 10.1186/s12943-022-01504-0 (PMC8764855; doi:10.1186/s12943-022-01504-0)
Supplement: Supplementary file 4 — Additional file 4. [file 12943_2022_1504_MOESM4_ESM.docx]

**Appendix Table 1.** Selection of gBRCA mutation patients in different cohorts.

| Clinical trial | Treatment group | No. of patients | BRCA status | |
| --- | --- | --- | --- | --- |
|  |  |  | Wildtype | Mutated |
| NCT01287624 | GP | 66 | 59 | 7 |
| NCT02341911 | GP | 1 | 1 | 0 |
| NCT02546934 | AP | 18 | 14 | 4 |
|  | GP | 29 | 28 | 1 |

**Appendix 1**.

**Step 1: specify predictors under the Cox model**

Based on the estimated coefficients in the multivariate Cox regression model included SNORD33 expression, liver metastasis and number of metastatic sites, we then build a prognostic nomogram to assign PFS probability at 2, 4, 6, 10, 12 and 18 months after first-line platinum treatment. The underlying Cox model is given by:

***Probability of event at time t*** *= S_0_(t) * exp (β_liver metastasis_*liver metastasis + β_SNORD33 expression_*SNORD33 expression +* *β_number of metastatic sites_ *number of metastatic sites) (1)*

Where S_0_(t) is called the baseline hazard function (possibly non-distributional). β is regression coefficients describing the effect of the predictors on the over probability. The right-hand side of the above equations specifies the underlying function of the model. The left-hand side of the equations is the predicted probability that is presented in a nomogram and communicated to patients.

*Related R software code for Cox model:*

*Library (survival)*

*fit<-cph(Surv(time,status) ~ Liver_metastasis + Number_of_metastatic_sites + SNORD33_expression, data = data, x=T, y=T, surv=T)*

**Step 2: construct the nomogram model**

In a nomogram, the point system work by ranking the effect estimates. In our nomogram, SNORD33 expression had the highest effect, thus it is converted into 100 points, with other remaining variables are assigned a smaller number of points proportional to their effect size. For example, the participant with liver metastasis would be given 66.7 points, which is equal to the ratio of *β_liver metastasis_ / β_SNORD33 expression<0.20_* multiplied by 100. The total points axis in Fig. 1n can go up to a maximum of 234.4 points.

*Related R software code for nomogram:*

*surv<-Survival(fit)*

*nom<-nomogram(fit, fun=list(function(x) surv(2,x), function(x) surv(4,x), function(x) surv(6,x), function(x) surv(8,x), function(x) surv(10,x), function(x) surv(12,x), function(x) surv(18,x)), funlabel=c("2-months PFS Probability","4-months PFS Probability", "6-months PFS Probability","8-months PFS Probability","10-months PFS Probability", "12-months PFS Probability", "18-months PFS Probability"))*

*plot(nom, xfrac=.4)*

**Step 3: calculate discrimination of nomogram: C index**

A concordance index (C index) was calculated to estimate the discriminative ability of the nomogram. A C index larger than 0.5 indicates prediction performance better than random guessing, with 1 indicating perfect discrimination [1]. In addition, the 1000 bootstrap resamples for internal validation was used.

*Related R software code for C-index of nomogram:*

*validate (fit, method = "boot", B=1000, dxy = T)*

**Step 4: calculate calibration of nomogram: calibration plot**

A visual calibration curve was plotted to evaluate the consistency between the nomogram-predicted probabilities and observed probabilities [2]. A perfect nomogram prediction would result in a 45-degree line, while the closer the predicted calibration curve is to the 45-degree line , the better the calibration of the nomogram was obtained.

*Related R software code for Calibration Curve of nomogram:*

*cal <- calibrate (fit, method = ‘boot’, B = 100)*

*plot(cal,xlim=c(0,1.0),ylim=c(0,1.0))*

**Appendix Figure 1**


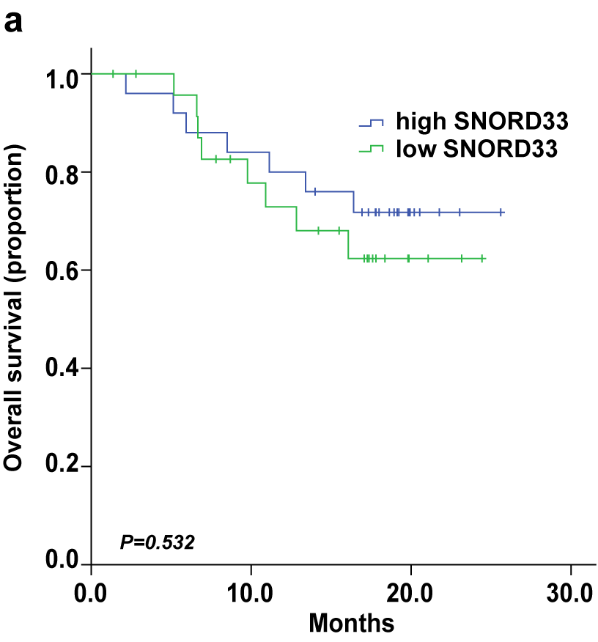


**Appendix Figure 1** Overall survival of non-small cell lung cancer (NSCLC) patients with low SNORD33 level tend to reduced.

Kaplan-Meier survival curves for overall survival (OS) (n=50). Cut-off threshold was median value; log-rank test. OS, *P*=0.532.

**Appendix Table 2.** Summary of methods, pros and cons of liquid biopsy-based biomarker in cancer.

| Biomarkers | Method of test | Advantage | Disadvantage |
| --- | --- | --- | --- |
| ctDNA | PCR, NGS | 1. Well established isolation procedure [3];  2. Suitable to identify genetic and epigenetic alterations [4];  3. High sensitivity [3]. | 1. Difficult to separate ctDNA from cell-free DNA [3];  2. Low levels of ctDNA in plasma and rapidly degraded in plasma with short half-life [4];  3. Poor specificity with increased level observed in some benign diseases [5];  4. High costs. |
| exosome | PCR | 1. Commercially available kits for isolation and testing;  2. Potentially to extract more information (e.g., protein) [6]; 3. Stable and accessible in plasma [7];  4. High sensitivity. | 1. The isolation, detection and characterization methods are challenging and require standardization [8];  2. High costs. |
| miRNA | PCR | 1.Commercial kits available for isolation;  2. Abundant in plasma [9];  3. Stable and accessible in plasma [10];  4. High sensitivity [10].  5. Low cost. | 1. Quantification and detection methods require standardization [9];  2. High variability in different population. |
| snoRNA | PCR | 1. Easy isolation procedure;  2. Higher abundance compared to miRNA in plasma [11];  3. Stable and accessible in plasma [12, 13];  4. High sensitivity [14].  5. Low cost. | 1. Quantification and detection methods require normalization;  2. High variability in different populations [15];  3. Commercial kits for collection need to be developed. |

Abbreviations: ctDNA, circulating tumor DNA; NGS, next generation sequencing.

References

1. Bandos AI, Rockette HE, Song T, and Gur D. Area under the free-response ROC curve (FROC) and a related summary index. Biometrics. 2009; 65(1): 247-56.

2. Harrell FE, Jr., Lee KL, and Mark DB. Multivariable prognostic models: issues in developing models, evaluating assumptions and adequacy, and measuring and reducing errors. Stat Med. 1996; 15(4): 361-87.

3. Bettegowda C, Sausen M, Leary RJ, Kinde I, Wang Y, Agrawal N, et al. Detection of circulating tumor DNA in early- and late-stage human malignancies. Sci Transl Med. 2014; 6(224): 224ra24.

4. Marques JF, Junqueira-Neto S, Pinheiro J, Machado JC, and Costa JL. Induction of apoptosis increases sensitivity to detect cancer mutations in plasma. Eur J Cancer. 2020; 127: 130-8.

5. Abbosh C, Birkbak NJ, and Swanton C. Early stage NSCLC - challenges to implementing ctDNA-based screening and MRD detection. Nat Rev Clin Oncol. 2018; 15(9): 577-86.

6. Halvaei S, Daryani S, Eslami SZ, Samadi T, Jafarbeik-Iravani N, Bakhshayesh TO, et al. Exosomes in Cancer Liquid Biopsy: A Focus on Breast Cancer. Mol Ther Nucleic Acids. 2018; 10: 131-41.

7. Siravegna G, Marsoni S, Siena S, and Bardelli A. Integrating liquid biopsies into the management of cancer. Nat Rev Clin Oncol. 2017; 14(9): 531-48.

8. Tellez-Gabriel M, Knutsen E, and Perander M. Current Status of Circulating Tumor Cells, Circulating Tumor DNA, and Exosomes in Breast Cancer Liquid Biopsies. Int J Mol Sci. 2020; 21(24).

9. Ozawa PMM, Jucoski TS, Vieira E, Carvalho TM, Malheiros D, and Ribeiro E. Liquid biopsy for breast cancer using extracellular vesicles and cell-free microRNAs as biomarkers. Transl Res. 2020; 223: 40-60.

10. Shimomura A, Shiino S, Kawauchi J, Takizawa S, Sakamoto H, Matsuzaki J, et al. Novel combination of serum microRNA for detecting breast cancer in the early stage. Cancer Sci. 2016; 107(3): 326-34.

11. Scott MS and Ono M. From snoRNA to miRNA: Dual function regulatory non-coding RNAs. Biochimie. 2011; 93(11): 1987-92.

12. Shang X, Song X, Wang K, Yu M, Ding S, Dong X, et al. SNORD63 and SNORD96A as the non-invasive diagnostic biomarkers for clear cell renal cell carcinoma. Cancer Cell Int. 2021; 21(1): 56.

13. Nossent AY, Ektefaie N, Wojta J, Eichelberger B, Kopp C, Panzer S, et al. Plasma Levels of snoRNAs are Associated with Platelet Activation in Patients with Peripheral Artery Disease. Int J Mol Sci. 2019; 20(23).

14. Kishikawa T, Otsuka M, Ohno M, Yoshikawa T, Takata A, and Koike K. Circulating RNAs as new biomarkers for detecting pancreatic cancer. World J Gastroenterol. 2015; 21(28): 8527-40.

15. Slaby O. Non-coding RNAs as Biomarkers for Colorectal Cancer Screening and Early Detection. Adv Exp Med Biol. 2016; 937: 153-70.
